# TMED3 promotes the progression and development of lung squamous cell carcinoma by regulating EZR

**DOI:** 10.1038/s41419-021-04086-9

**Published:** 2021-08-24

**Authors:** An Xie, Xinping Xu, Peng Kuang, Ling Zhang, Feng Yu

**Affiliations:** 1 Jiangxi Institute of Urology, The First Affiliated Hospital of Nanchang University, 17 Yong Wai Zheng Street, Nanchang City, Jiangxi Province China; 2 Jiangxi Institute of Respiratory Disease, The First Affiliated Hospital of Nanchang University, 17 Yong Wai Zheng Street, Nanchang City, Jiangxi Province China; 3 Department of Oncology, The First Affiliated Hospital of Nanchang University, 17 Yong Wai Zheng Street, Nanchang City, Jiangxi Province China

**Keywords:** Non-small-cell lung cancer, Cell biology

## Abstract

Lung squamous cell carcinoma (LUSC) has a poor clinical prognosis and lacks effective targeted therapy. The transmembrane emp24 trafficking protein 3 (TMED3) belongs to the TMED family, which is responsible for the transport of intracellular proteins. This study was to explore the clinicopathological significance and biological effects of TMED3 in LUSC. Expression of TMED3 in LUSC was detected by immunohistochemical (IHC). The loss-of-function assays were used to investigate the effects of TMED3 on proliferation, apoptosis, cell cycle, and migration of LUSC cells. The influence of TMED3 knockdown on tumor growth in vivo was evaluated by mice xenograft models. In addition, the downstream target of TMED3 was recognized by RNA sequencing and Ingenuity Pathway Analysis (IPA). Moreover, TMED3 was upregulated in LUSC tissue, which was positively correlated with pathological grade. TMED3 knockdown was involved in the regulation of LUSC cell function, such as inhibition of proliferation, reduction of colony formation, induction of apoptosis, and reduction of migration. TMED3 knockdown induced abnormalities in apoptosis-related proteins in LUSC cells. In addition, the inhibition of cell migration by TMED3 knockdown was achieved by regulating EMT. Mechanically, EZR was considered as a potential target for TMED3 to regulate the progress of LUSC. Inhibition of EZR can inhibit the progression of LUSC, and even reduce the promoting effects of TMED3 overexpression on LUSC. In conclusion, TMED3 promoted the progression and development of LUSC by EZR, which may be a novel therapeutic target for LUSC.

## Introduction

Lung cancer has become the leading cause of death, with more deaths than colorectal, breast, prostate, and pancreatic cancer combined, and nearly 1.8 million people are diagnosed with lung cancer each year [1]. Lung squamous cell carcinoma (LUSC) is a subtype of non-small cell cancer and accounts for ~40% of all lung cancer, which based on age or extent of tobacco exposure [2]. Despite advances in diagnosis and treatment in recent years, 5-year overall survival rate of LUSC remains low [3]. Currently, the first-line treatment of LUSC is platinum-based chemotherapy coupled, immunotherapy or a combination of both. The second-line treatment is immunotherapy, single-agent chemotherapy, or with anti-angiogenesis or anti-EGFR tyrosine kinase inhibitors. Although these treatments have been shown to be clinically effective, long-term application is prone to drug resistance [4, 5]. In addition, Whole Genome Sequencing confirmed that this disease has a high mutation rate [6]. Accordingly, targeted therapy of LUSC has become a hot spot. Recently, Yuan et al., identified NSD3 as a principal 8p11–12 amplicon-associated oncogenic driver in LUSC, and suggested that NSD3-dependency renders LUSC therapeutically vulnerable to bromodomain inhibition [7]. Unfortunately, the effect of targeted therapy for LUSC has not met the requirements of patients [8]. Thus, the poor clinical prognosis and the lack of effective targeted drugs of LUSC suggested that we should continue to explore its molecular mechanism of development and progression to ascertain promising appropriate targets.

The transmembrane emp24 domain/p24 (TMED) protein mainly exists as monomer, dimer, oligomer and isomer in eukaryotes, which plays a role in Golgi kinetics and intracellular protein transport [9]. Cytoplasmic N-terminal and TMD sequences mediate the transport of carrier membrane proteins to the plasma membrane via the endoplasmic reticulum-Golgi-trans Golgi network pathway [10]. Transmembrane emp24 protein transport domain containing 3 (TMED3) belongs to a family of TMED, has also been named C15orf22 or P26, which is involved in protein vesicle transport and innate immune signal transmission [11]. The role of TMED3 in cancer is controversial. For example, TMED3 inhibits the distant metastasis of colon cancer [12], but promotes tumor progression of liver cancer and breast cancer [13, 14]. Recently, Yang et al., suggested that knockdown of TMED3 inhibited cell viability and migration, and enhanced apoptosis in chordoma cells [15]. Besides, TMED3 is a potential prognostic factor for clear cell renal cell carcinoma [16]. However, the role of TMED3 in LUSC and the underlying molecular mechanisms have not been elucidated.

The key role of TMED3 in LUSC was explored in this study. The differential expression of TMED3 between LUSC tissues and normal tissues was detected, revealing that TMED3 was highly expressed in LUSC. Subsequent in-depth exploration indicated that knockdown of EZR can inhibit the progression of LUSC and reduce the promotion effects of TMED3 overexpression in LUSC. Therefore, TMED3 may be identified as a new therapeutic target in LUSC treatment strategy.

## Materials and methods

### Cell culture

LUSC cell lines including EBC-1, NCI-H520, and SK-MES-1 were obtained from the cell bank of the Shanghai Academy of Sciences in China. They were cultured with RPMI-1640 medium (Carlsba Life Technologies, USA) at 37 °C, 5% CO_2_, and supplemented with 10% fetal bovine serum (FBS, Life Technologies, Inc.). In addition, these cell lines were tested for mycoplasma contamination and maintained in prophylactic dose of plasmocin.

### Target RNA interferes with the preparation of lentiviral vector

The lentivirus-mediated shRNA vector BR-V108 system was designed, constructed, packaged, and purified by Shanghai Bioscienceres (Shanghai, China). Human TMED3-targeting shRNA, EZR-targeting shRNA were designed (Table S1) and cloned into lentiviral vector BR-V108. Fluorescence microscope (Olympus, Tokyo, Japan) subsequently demonstrated the transfection efficiency of lentivirus-transfected cells. Finally, the knockdown efficiency of target gene was detected by qPCR and western blot. The recombinant lentiviruses expressing TMED3 shRNA, EZR shRNA, overexpressing TMED3, and BR-V108 vector infected EBC-1, NCI-H520, and SK-MES-1 cells, which were named as shTMED3, shEZR, TMED3, TMED3+shEZR, NC(OE+KD), shCtrl, and Control, respectively.

### Immunohistochemical (IHC) staining

After the patient was diagnosed based on clinical and pathological evidence, the specimens were immediately frozen and stored in liquid nitrogen tanks. All patients signed informed consent to use clinical data for research purposes. Ninety cases of paraffin-embedded LUSC tissues were purchased from Shanghai Outdo Biotechnology Co., Ltd. After tissue sections were deparaffinized, repaired, and blocked with citric acid antigen, they were incubated with antibody TMED3 and EZR (Table S2), respectively, overnight. After elution with PBS, secondary antibody IgG was added and incubated. Tissue sections were stained with DAB and hematoxylin for visualization. Finally, images were captured under a light microscope and scores were assessed by German immune response score [17].

### Quantitative real time PCR (qPCR)

Total RNA was extracted from the EBC-1, NCI-H520 and SK-MES-1 cells infected with TMED3 (over-expression), shEZR, TMED3+shEZR, and the corresponding negative controls using TRIzol^®^ (Thermo Fisher Scientific, Waltham, MA, USA), followed by reverse transcription using M-MLV reverse transcriptase and RNase inhibitor (Promega Corporation, Madison, Wisconsin, USA) according to the manufacturer’s protocol. Primer sequence information was shown in the Table S3. The qPCR was performed using SYBR Master Mix (Takara, Japan), where GAPDH was used as the internal control. Finally, the 2^−ΔΔCq^ method was used for quantification and dissolution curve was drawn.

### Western blot analysis

Protein was extracted from the cells EBC-1, NCI-H520, and SK-MES-1 infected with TMED3 over-expression, shEZR, TMED3+shEZR and the corresponding negative controls) using lysis buffer (Nanjing KeyGen Biotech, Nanjing, Jiangsu, China), and protein concentration is measured using the BCA protein assay kit (Takara, Otsu, Japan). Total proteins are separated by SDS-PAGE (10%) and transferred to poly (vinylidene fluoride) membrane. The membrane was blocked in buffered saline Tris (TBST, Tween 20) and incubated with the following primary antibodies: TMED3, EZR, Akt, p-Akt, Cyclin D1, CDK6, PIK3CA, and GAPDH (loading control) (Table S2). After washing the membrane in TBST, the membrane was conjugated with horseradish peroxidase-conjugated goat anti-mouse immunoglobulin secondary antibody IgG (Table S2). Bands were observed using ECL-Plus™ (Thermo Fisher Scientific, NY, USA) according to the manufacturer’s protocol.

### MTT assay

The effects of TMED3 or EZR knockdown on cell proliferation were analyzed by MTT assay in the cited literature [18]. Cells (EBC-1, NCI-H520 and SK-MES-1) infected with TMED3 (over-expression), shEZR, TMED3+shEZR, and the corresponding negative controls for 72 h were seeded in 96-well plate (2 × 10^3^ cells/well). Total 20 µL of 5 mg/ML MTT (Genview, Beijing, China, Cat. # JT343) was added into each well at 37 °C. After 4 h, the medium was discarded, and DMSO (Shanghai Test Chemical Reagent Co., Ltd., Shanghai, China) was added to each well to dissolve the methylaz crystals. Optical density (OD490 nm) was detected through a multifunctional microplate reader (Tecan Group, Ltd., Mannedorf, Switzerland).

### Colony formation

Cells NCI-H520 and SK-MES-1 infected with TMED3 (over-expression), shEZR, TMED3+shEZR and the corresponding negative controls were trypsinized, resuspended, counted, and seeded in six-well plate (2 ml/well) and incubated for 8 days to form colonies. The cells were washed with PBS, fixed with paraformaldehyde, stained with Giemsa, washed again, and photographed with a digital camera. The number of colonies (>50 cells/colonies) was counted under a microscope (Micro Publisher 3.3 RTV; Olympus, Tokyo, Japan). All determinations were repeated three times.

### Celigo cell counting assay

EBC-1, NCI-H520, and SK-MES-1 cells (2 × 10^3^ cells/well) after infection 72 h with TMED3 (over-expression), shEZR, TMED3+shEZR and the corresponding negative controls were seeded in 96-well plate. After plating, the number of cells was evaluated using a Celigo^®^ image cytometer (Nexcelom, Lawrence, Mass., USA) by scanning green fluorescence every day for 5 days at room temperature.

### Flow cytometry

Cell apoptosis was analyzed by flow cytometry according to the methods provided in the literature [19]. EBC-1, NCI-H520, and SK-MES-1 cells infected with TMED3 (over-expression), shEZR, TMED3+shEZR, and the corresponding negative controls were trypsinized, resuspended. Annexin V-APC was added to stain in the dark for 15 min, and the percentage of cellular phase was determined by flow cytometry to evaluate the rate of apoptosis.

### Transwell invasion assay

According to the method provided in the literature [20], the suspension of cells (EBC-1, NCI-H520, and SK-MES-1) infected with TMED3 (over-expression), shEZR, TMED3+shEZR, and the corresponding negative controls were first adjusted to 5 × 10^5^ cells/mL, and then the cells were seeded into Transwell (Corning, NY, USA). The cells were fixed with 4% paraformaldehyde and then stained with 0.1% crystal violet solution. Finally, the cells passing through the filter were photographed by an inverted fluorescence microscope.

### Wound-healing migration assay

Citing the method in the literature [21], after infection 72 h of EBC-1, NCI-H520 and SK-MES-1 cells (TMED3 overexpression, shEZR, TMED3+shEZR, and corresponding negative controls) (2 × 10^3^ cells /well) were scraped a monolayer at the scheduled time (0, 4, 8 h) for wound-healing assay and under 5% CO_2_ incubated at 37 °C.

### Human apoptosis antibody array

Differentially expressed apoptosis-related proteins in NCI-H520 cells induced by TMED3 knockdown were evaluated by human apoptosis antibody array (RayBio, Norcross, GA, USA). First, the membrane was placed in a petri dish, and cell lysate was added to each well, and gently shaken at 4 °C overnight. After that the membrane was washed and incubated with lyophilized biotinylated antibody. The excess molecules were washed off and then incubated with horseradish peroxidase-conjugated streptavidin. Gelpro Analyzer software (Media Cybernetics, Rockville, MD, USA) was used to analyze protein expression levels.

### Animal xenograft model

The animal experiments performed here have been approved by the Animal Protection and Use Committee of The First Affiliated Hospital of Nanchang University. Nude mice (BALB/c males, 4 weeks old) were purchased from Shanghai Experimental Animal Co., Ltd. (Shanghai, China) and are pathogen-free. The 20 mice were randomly divided into two groups (shCtrl group and shTMED3 group) before the experiments. EBC-1 cells (4 × 10^6^) suspended in PBS were injected subcutaneously into the mice through the right armpit to construct a mouse xenograft model. Mice were cultured for another 20 days after injection, and data were collected starting from 10 days after injection. The tumor length and width and the weight of the mice were measured every other day thereafter. Finally, the mice were sacrificed by injection of sodium pentobarbital, and the tumors were photographed and weighed. After tumor tissues were excised from the sacrificed mice, they were repaired and blocked with citrate antigen. Antibody Ki67 (Table S2) was added to shTMED3 or shCtrl, respectively. After PBS elution, secondary antibody IgG (Table S2) is added and incubated at room temperature. Tissue sections were stained first with DAB and then with hematoxylin. Finally, the images were collected with an optical microscope and analyzed.

### RNA sequencing

In short, NCI-H520 cells RNA was extracted and tested for quality as described above. The library for RNA sequencing was constructed from the TruSeq Stranded mRNA LT Sample Preparation Kit (Illumina, San Diego, California, USA) according to the instructions of manufacturer, and then scan it by Affymetrix Scanner 3000 (Affymetrix, Santa Clara, California, USA). DEGs were determined between the two groups (shCtrl group and shTMED3 group) based on thresholds of absolute fold change > 2 and FDR < 0.05. IPA (Qiagen, Hilden, Germany) based on all DEGs to analyze rich functional annotations. An absolute value of the *Z*-score > 2 was considered significant.

### Immunofluorescence detection

For co-localization, NCI-H520 cells were transfected with shCtrl or shEZR and incubated for 48 h. Subsequently, the cells were fixed in 4% paraformaldehyde for 20 min, followed by incubation with 0.25% Triton X-100 for 40 min and finally blocking in 5% bovine serum albumin for 1 h. Next, the cells were incubated with rabbit antibody against TMED3/EZR and mouse anti-flag at 4 °C overnight and then exposed to fluorochrome-labeled antibodies for 1 h at 25 °C (Alexa Fluor 488 donkey anti-rabbit IgG and Alexa Fluor 568 goat anti-mouse IgG). DAPI staining was performed for 5 min. Cells were imaged using a confocal laser scanning microscope (PerkinElmer, Waltham, MA, USA).

### Statistical analysis

Statistical analysis was performed using SPSS 20.0 (SPSS, Chicago, Illinois, USA) and GraphPad Prism 6.0 (GraphPad, La Jolla, California, USA). Student *t* test and Chi-square test were used in the data analysis. All IHC, qPCR, western blot, cellular functions assays, and IPA were conducted ≥ 3 times to ensure reproducibility. The data of each experiment were shown as mean ± SD (*n* ≥ 3). *P* < 0.05was considered statistically significant. An absolute value of *Z*-score > 2 is considered significant in IPA.

## Results

### The expression of TMED3 is upregulated in LUSC

**Fig. 1:**
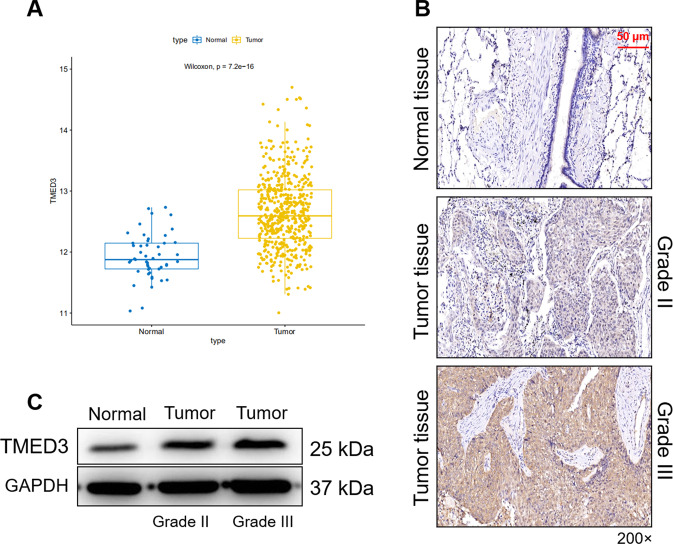
TMED3 is highly expressed in LUSC. **A** Expression profiles of TMED3 were analyzed based on 501 tumor samples and 49 normal samples in the TCGA. **B** The expression of TMED3 in the normal and tumor samples was detected by IHC (200 × magnification: scale bar = 50 μm). **C** The protein expression of TMED3 in normal and tumor tissues was detected by western blot.

Expression profiles were analyzed based on 501 tumor samples and 49 normal samples in the TCGA (The Cancer Genome Atlas) database, finding that the expression level of TMED3 in tumor was significantly higher than that in normal (*P* < 0.01) (Fig. 1A). In order to further clarify the role of TMED3 in LUSC, we first detected the expression of TMED3 in clinical LUSC patients by immunohistochemistry. As shown in the representative image in Fig. 1B, the signal intensity of TMED3 in tumor tissue was significantly stronger than that in normal tissue. Statistical analysis in Table 1 showed that high expression of TMED3 accounted for 75.9 in tumor tissues, while there was no high expression of TMED3 in adjacent normal tissues. Consistently, results of western blot indicated that protein expression of TMED3 was highly expressed in tumor tissue. Especially, the expression of TMED3 in tumor grade III was higher than that in tumor grade II (Fig. 1C). In addition, significant positive correlation between the expression of TMED3 and the grade, T Infiltrate in the pathological data was exposed through Mann-Whitney *U* analysis (Table 2). Subsequent multivariate cox regression analysis indicated that the expression levels of TMED3 were positively correlated with grade and T Infiltrate (Table 3). Kaplan–Meier analysis showed that although TMED3 expression was not significantly associated with survival in LUSC patients, LUSC patients with high TMED3 expression had a shorter survival (Fig. S1A). Taken together, expression of TMED3 was upregulated in LUSC.

**Table 1:** Expression patterns in lung squamous cell carcinoma tissues and para-carcinoma tissues revealed in immunohistochemistry analysis.

TMED3 expression	Tumor tissue		Para-carcinoma tissue		*p* value
	Cases	Percentage	Cases	Percentage	
Low	20	24.1%	89	100%	<0.001
High	63	75.9%	0	/

**Table 2:** Relationship between TMED3 expression and tumor characteristics in patients with lung squamous cell carcinoma.

Features	No. of patients	TMED3 expression		*P* value
		low	high	
All patients	83	20	63	
Age (years)				0.950
<64	41	10	31	
64	40	10	30	
Gender				0.196
Male	78	20	58	
Female	5	0	5	
Grade				0.023
II	63	19	44	
III	20	1	19	
AJCC stage				0.355
1	25	8	17	
2	23	5	18	
3	32	7	25	
4	1	0	1	
T Infiltrate				0.214
T1	11	6	5	
T2	49	9	40	
T3	16	4	12	
T4	4	1	3	
lymphatic metastasis (*N*)				0.626
N0	45	12	33	
N1	12	2	10	
N2	8	2	6	
Tumor size				0.122
<4.5 cm	36	12	24	
≥4.5 cm	44	8	36

**Table 3:** Relationship between TMED3 expression and tumor characteristics in patients with lung squamous cell carcinoma.

		TMED3
Grade	Pearson correlation	0.252
	Significance (double-tailed)	0.022
	*N*	83

### Knockdown of TMED3 inhibits the progression of LUSC in vitro

**Fig. 2:**
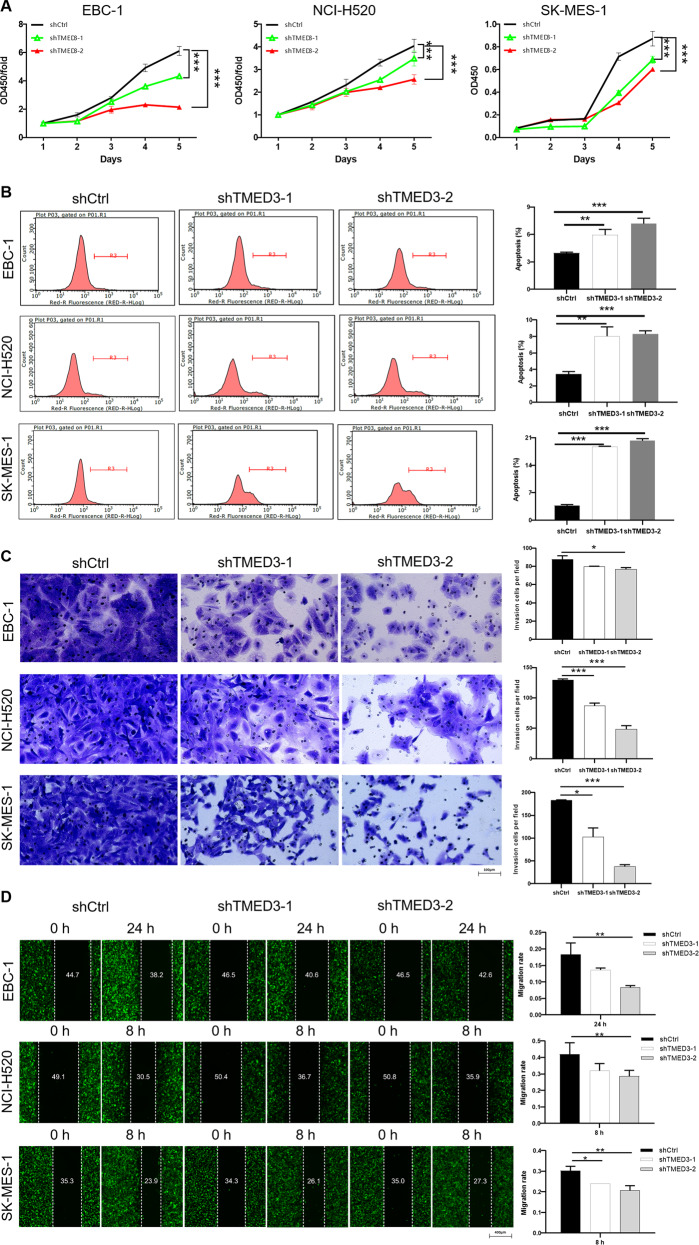
The effect of TMED3 on cell proliferation, apoptosis, invasion, migration, and apoptosis/EMT-related proteins in LUSC. **A** MTT assay was used to evaluate the effect of TMED3 knockdown on proliferation of EBC-1, NCI-H520, and SK-MES-1 cells. **B** The effect of TMED3 knockdown on apoptosis of EBC-1, NCI-H520, and SK-MES-1 cells was evaluated by flow cytometry. **C** The changes of TMED3 knockdown on the invasion of EBC-1, NCI-H520, and SK-MES-1 cells were detected by the Transwell experiment (200× magnification: scale bar = 100 μm). **D** The would-healing assay was used to evaluate the effect of TMED3 knockdown on migration of EBC-1, NCI-H520, and SK-MES-1 cells.

In order to further explore the impacts of TMED3 on the progression of LUSC, EBC-1, NCI-H520, and SK-MES-1 cells were used to construct TMED3 knockdown cell models. Lentivirus-expressed short hairpin RNA (shRNA) was used as the shTMED3 group and scrambled lentivirus was the negative control group (shCtrl). Knockdown efficiency of TMED3 was evaluated by qPCR (Fig. S1B) and western blot analysis (Fig., S1C), indicating that depletion of TMED3 in these cell lines exceeded 60%. Subsequently, MTT (Fig. 2A), flow cytometry (Fig. 2B), Transwell (Fig. 2C), and wound-healing assays (Fig. 2D) were used to evaluate the effects of TMED3 knockdown on proliferation, apoptosis, invasion, and migration of LUSC cells. As illustrated in Fig. 2A, the proliferation ability of EBC-1, NCI-H520 and SK-MES-1 cells decreased significantly after TMED3 knockdown. Inhibitory effect of shTMED3-2 group on cell proliferation was higher than that of shTMED3-1 group. Moreover, decreased expression of TMED3 resulted in increased apoptotic sensitivity, especially the shTMED3-2 group was stronger than the shTMED3-1 group (Fig. 2B). Figure 2C showed that TMED3 knockdown resulted in a decrease in the number of invaded cells. Not surprisingly, knockdown of TMED3 dramatically inhibited the migration of EBC-1, NCI-H520, and SK-MES-1 cells (Fig. 2D). We performed two shRNA-mediated depletion experiments through functional testing, which excluded off-target effects. Thus, these results indicated that knockdown of TMED3 dramatically inhibited the proliferation, migration and promoted the cell apoptosis in LUSC cells. Furthermore, human apoptosis antibody array was applied to detect the differential expression of apoptosis-related proteins induced by TMED3 knockdown in NCI-H520 cells. The results showed that the expression levels of proteins Bcl-2, clAP-2, IGF-1sR, and Livin were significantly downregulated. On the contrary, Caspase3, Caspase8, IGFBP-6, SMAC expression level was significantly upregulated (Fig. S2A). Subsequently, western blot (Fig. S2B) was performed to detect the expression levels of several epithelial–mesenchymal transition (EMT) biomarkers in NCI-H520 and SK-MES-1. The results displayed that knockdown of TMED3 upregulated expression of E-cadherin, while downregulating of N-cadherin, Vimentin, and Snail protein expression. In general, TMED3 was required for the proliferation, apoptosis, invasion, migration, and EMT of LUSC cells.

### Knockdown of TMED3 inhibited tumor growth of LUSC in vivo

**Fig. 3:**
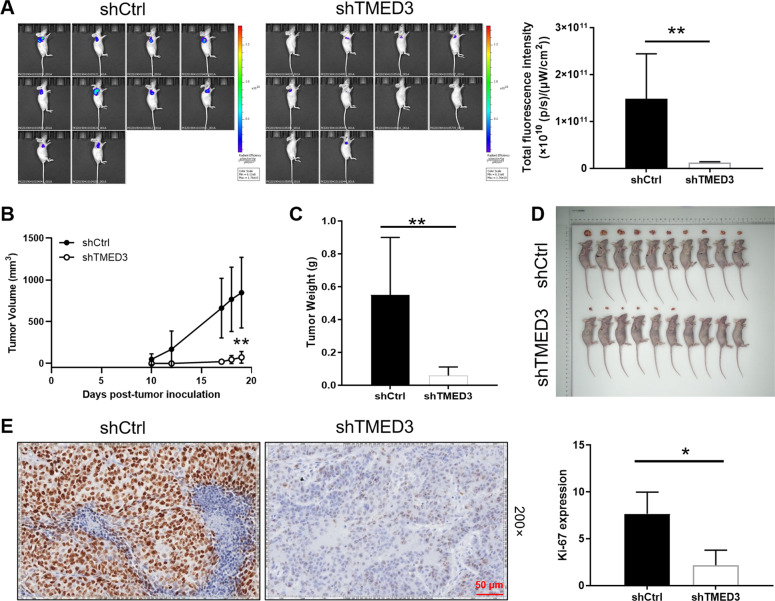
Knockdown of TMED3 inhibits tumor growth in vivo. **A** The total fluorescence intensity of tumors in shCtrl group and shTMED3 group. **B** The volume of tumors in shCtrl group and shTMED3 group was measured and calculated during experiments. **C** The weight of tumors in shCtrl group and shTMED3 group was measured after the sacrifice of mice. **D** The photo of xenograft tumors removed from the mice models in shCtrl and shTMED3 groups. **E** The Ki-67 index of the removed tumors was evaluated by IHC analysis (200 × magnification: scale bar = 50 μm).

To further verify the role of TMED3 in LUSC, we constructed a mouse xenograft model by subcutaneous injection of EBC-1 cells with or without TMED3 knockdown. The fluorescence imaging indicated that the fluorescence intensity of the shTMED3 group was notably lower than that of the shCtrl group (*P* < 0.01), suggesting that the tumorigenicity of the shTMED3 group was weaker than that of the control group (Fig. 3A). Compared with the shCtrl group, the tumor growth of LUSC in shTMED3 group was inhibited, tumor volume was smaller (*P* < 0.01) (Fig. 3B), tumor weight was reduced (*P* < 0.01) (Fig. 3C), and the resected tumors were shown in Fig. 3D. In addition, Ki67 index was further evaluated by IHC analysis. As shown in Fig. 3E, expression of Ki67 in shTMED3 group was lower than that in shCtrl group. The results in vivo demonstrated that TMED3 knockdown had an inhibitory effect on the proliferation of cancer cells, these results indicate the promotive role of TMED3 in LUSC.

### Exploration of downstream molecular mechanism of TMED3 in LUSC cells

**Fig. 4:**
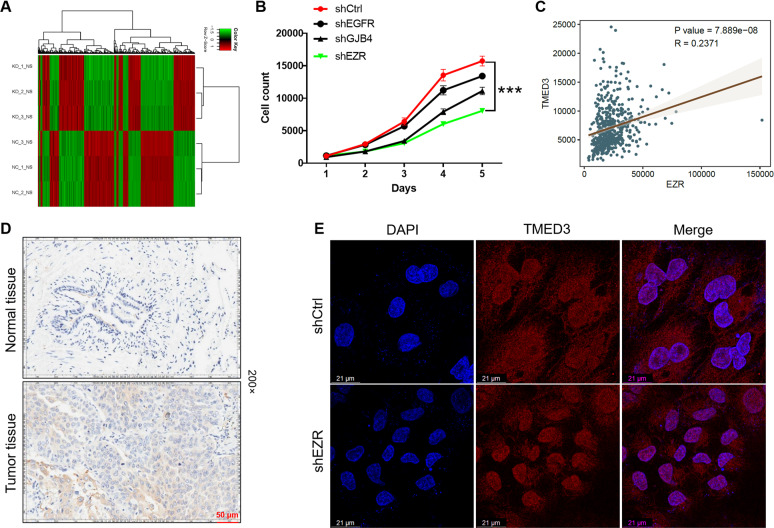
Exploration of underlying mechanism of TMED3 in LUSC. **A** NCI-H520 cells transfected with lentivirus shCtrl (*n* = 3) or shTMED3 (*n* = 3) were RNA sequenced to determine DEGs. **B** MTT assay was used to evaluate the effect of EGFR, EZR, and GJB4 knockdown on proliferation of NCI-H520 cells. **C** Pearson correlation analysis were performed to examine the relationship between TMED3 and EZR. **D** The expression of EZR in normal and tumor tissues was detected by IHC analysis (200× magnification: scale bar = 50 μm). **E** Co-localization was observed by immunofluorescence. Scale bar = 21 μm.

To further elucidated the effects of TMED3 knockdown on downstream mechanisms in LUSC, shTMED3 (*n* = 3, as experimental group) or shCtrl (*n* = 3, as control group) infected NCI-H520 cells are used for RNA profiling analysis by RNA sequencing. In general, according to |Fold Change| value ≥ 1.5 and *P* value < 0.05, a total of 1142 Differentially Expressed Genes (DEGs) are identified, of which 551 genes were upregulated and 591 genes were downregulated (Fig. 4A). Inventive Pathway Analysis (IPA) was performed on canonical signaling pathways, disease or function, and interaction network analysis to identify gene enrichment induced by TMED3 knockdown (Fig. S3A–C). Based on the bioinformatics, we significantly downregulated DEGs in the shTMED3 group for qPCR verification (Fig. S3D). The most significantly downregulated candidate genes, including EGFR, EZR, GJB4 were selected for western blot analysis in NCI-H520 cells (Fig. S3E). In addition, lentivirus was used to deliver shRNAs into NCI-H520 cells to knockdown these genes (EGFR, EZR, GJB4), respectively. As shown in Fig. 4B, the inhibitory effect of shEZR group on proliferation was the most significant compared with other groups (*P* < 0.001). EZR was the most relevant to the proliferative phenotype of NCI-H520 cells. Pearson correlation analysis was performed to examine the relationship between TMED3 and EZR, indicating that TMED3 and EZR were significantly positively correlated (Fig. 4C). Moreover, the IHC analysis further validated above proposal, indicating that significant upregulation of EZR expression in LUSC tissues, compared with adjacent normal tissues (Fig. 4D). Furthermore, TMED3 is a transmembrane protein. Figure 4E showed the subcellular localization of TMED3 after EZR knockdown by immunofluorescence detection. We found that the subcellular localization of TMED3 was almost unchanged but its expression level was reduced. Collectively, EZR was preliminarily identified as a downstream target of TMED3 in NCI-H520 cells.

### Knockdown of EZR alleviated the promotion effect of TMED3 overexpression on LUSC

**Fig. 5:**
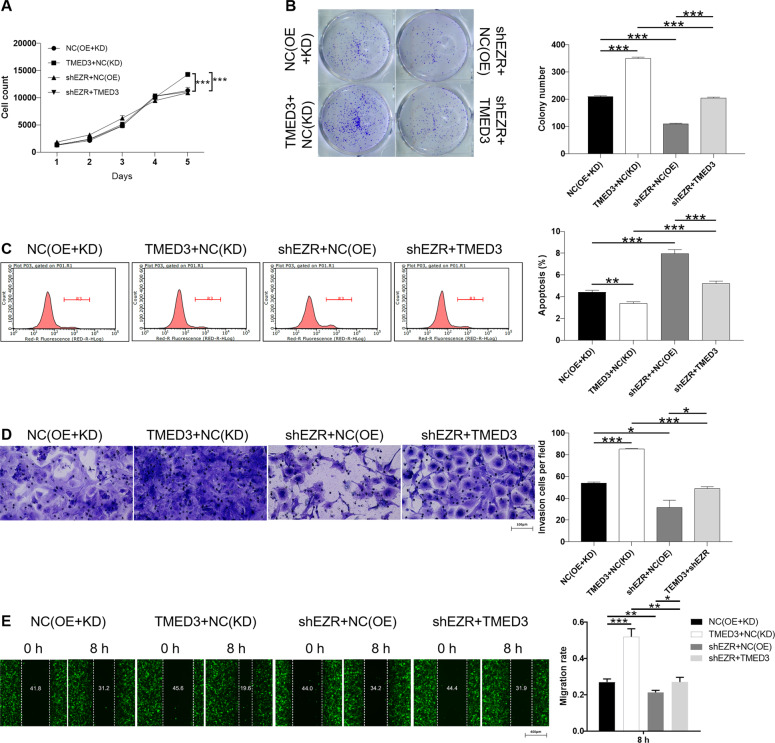
EZR knockdown alleviate the effects of TMED3 overexpression on LUSC cells. The effects of overexpression of TMED3 or knockdown of EZR on proliferation (**A**), colony formation (**B**), apoptosis (**C**), invasion (200 × magnification: scale bar = 100 μm) (**D**), and migration (**E**) of NCI-H520 cells were investigated. NC(OE+KD) was the cells transfected with empty plasmid, as negative control; TMED3+NC(KD) was the cells transfected with lentivirus TMED3 and NC-shEZR for overexpressing TMED3; shEZR+NC(OE) was the cells transfected with lentivirus shEZR and NC-TMED3 for downregulating EZR; shEZR+TMED3 was the cells transfected with shEZR and TMED3 for simultaneously downregulating EZR and overexpressing TMED3.

A series of loss/gain-of-function assays were conducted to further determine the role of TMED3 and downstream EZR in LUSC cells. Firstly, functional experiments were conducted following the construction of EZR knockdown in NCI-H520 cell. These results of cell experiments indicated that knockdown of EZR dramatically inhibited the proliferation (*P* < 0.001), invasion (*P* < 0.05), migration (*P* < 0.01), and promoted the cell apoptosis (*P* < 0.001) (Fig. 5A-E). EZR knockdown inhibited the progression of LUSC, which exhibited a similar effect to TMED3. On the other hand, overexpression of TMED3 can promote proliferation (*P* < 0.001) (Fig. 5A), increase colony formation (*P* < 0.001) (Fig. 5B), inhibit apoptosis (*P* < 0.01) (Fig. 5C), strengthen invasion (*P* < 0.01) (Fig. 5D) and induce migration (*P* < 0.001), (Fig. 5E) of NCI-H520 cells. Interestingly, these results were exactly the opposite of knockdown effect of TMED3. Meanwhile, the NCI-H520 cells with simultaneously upregulated TMED3 and downregulated EZR (TMED3+shEZR) were established (Fig. S3F). downregulation of EZR in TMED3 overexpressed NCI-H520 cells can alleviate the TMED3 overexpression induced promotion of proliferation (Fig. 5A), colony formation (Fig. 5B), invasion (Fig. 5D), migration (Fig. 5E), and inhibit apoptosis (Fig. 5C). Therefore, recovery experiments demonstrated that EZR knockdown could restore the promoting effect of TMED3 overexpression on the progression of LUSC.

## Discussion

Due to the lack of clinical symptoms or effective biomarkers, most patients with LUSC are at an advanced stage when they are first diagnosed [22]. In order to improve the diagnosis and prognosis of LUSC, it is urgent to clarify the molecular mechanism of LUSC and to identify new reliable biomarkers and therapeutic targets. In this study, the clinical significance of TMED3 and its role in LUSC were investigated. The expression of TMED3 in LUSC was identified by IHC analysis. The effects of endogenous TMED3 on the development and progression of LUSC were explored by constructing cell models of TMED3 knockdown and overexpression in LUSC cell lines. These results demonstrated that knockdown of TMED3 significantly inhibited proliferation, colony formation, invasion, and migration ability, and promoted apoptosis. On the contrary, overexpression of TMED3 showed the opposite effect. In addition, in vivo mice xenograft model further illustrated the inhibition of LUSC tumor growth by TMED3 knockdown.

Additionally, we found that apoptosis induced by TMED3 knockdown in NCI-H520 cells was a combination of a series of apoptosis-related proteins. For instance, Bcl-2, clAP-2, IGF-1sR, and Livin expression levels were downregulated, Caspase3, Caspase8, IGFBP-6, and SMAC expression levels were upregulated. Previous studies suggested that Bcl-2 and Bax proteins were members of the Bcl-2 family and regulated the intrinsic apoptotic pathway [23]. Li et al. pointed that Livin subtype is expressed differently in different types of lung cancer tissues, which may lead to corresponding cancer development, and Livin level was negatively correlated with Caspase3, which may be due to the anti-apoptotic effect of Livin [24]. Moreover, Cao et al. clarified that induction of apoptosis in LUSC cells can significantly increase the reduction of XIAP and Survivin proteins and the simultaneous release of SMAC, EndoG, and AIF in mitochondria [25]. Therefore, combining the above conclusions, it can be concluded that the apoptosis induced by TMED3 knockdown in LUSC cells was achieved through the regulation of a series of apoptosis-related proteins.

EMT is a developmental process that promotes invasion and metastasis in various types of tumors [26]. During EMT, E-cadherin, N-cadherin, Vimentin, and Snail are the most commonly detected epithelial and mesenchymal markers, respectively [27]. Therefore, the present study assessed a series of proteins associated with EMT using western blot analysis. We found that knockdown of TMED3 upregulated expression of E-cadherin, while downregulating of N-cadherin, Vimentin, and Snail protein expression in NCI-H520 cells. These findings suggesting that TMED3 knockdown can inhibit EMT and recognized the key role of EMT in invasion and migration.

Moreover, the exploration of downstream of TMED3 determined that EZR is a potential target by a genome-wide expression profiling analysis and IPA. EZR (ezrin) is a member of the Ezrin–Radixin–Moesin family. It regulates the cytoskeleton as a regulator of signal molecules and participates in many processes necessary for cell growth, such as adhesion, migration, cell division, and surface structure formation [28, 29]. Studies have confirmed that EZR is overexpressed in many human cancers and involved in many aspects of tumor cell migration [30–32]. Zhang et al. demonstrated that EZR plays an important role in promoting mobility and invasiveness of human esophageal squamous cell carcinoma cells [32]. Besides, Zhang et al. supported EZR is upregulated in breast cancer and can be used as a potential biomarker for overall survival [33]. Liu et al. proposed LncRNA EZR-AS1 silencing can inhibit the proliferation, invasion, and migration of colorectal cancer cells by blocking transforming growth factor β signaling [34]. Arai et al. verified that EZR-ROS1 plays a vital role in the development of non-small cell lung cancer carrying the fusion gene [35]. While, this study elucidated the inhibitory effect of EZR knockdown on LUSC similar to TMED3 by detecting proliferation, colony formation, apoptosis, and migration. More importantly, downregulating EZR may weaken the role of TMED3 overexpression and promote LUSC to a certain extent, suggesting that TMED3 may regulate the development of LUSC by affecting EZR.

In summary, this study indicated that TMED3 expression was upregulated in LUSC tissues and its expression positively correlated with tumor progression. Further research clarified that overexpression/knockdown of TMED3 can promote/inhibit LUSC cell proliferation, colony formation, and migration, and inhibit/promote apoptosis. We also declared that knockdown of TMD3 induced apoptosis in LUSC cells was co-regulated by a series of apoptosis-related proteins (Bcl-2, clAP-2, IGF-1sR, and Livin expression levels are significantly downregulated, Caspase3, Caspase8, IGFBP-6, and SMAC expression levels are significantly upregulated). Moreover, TMED3 knockdown can inhibit EMT and recognized the key role of EMT in invasion and migration. Besides, downstream of TMED3 was explored and EZR was identified as a potential target by RNA sequencing followed by IPA. Furthermore, our study supported that knockdown of EZR can inhibit the development of LUSC in vitro and reduce the promotion of LUSC induced by TMED3 overexpression. Thus, TMED3 may be a potential molecular target for the treatment of LUSC in the future. Our findings provide an insight into understanding the development and progression of LUSC, but specific mechanisms such as the downstream signal pathway of TMED3 to regulate LUSC need further exploration.

## Supplementary information

Supplementary tables

Supplementary figure legends

Figure S1

Figure S2

Figure S3

aj-checklist

cddis-author-contribution-form

## Data Availability

The datasets used and/or analyzed during the current study are available from the corresponding author on reasonable request.
