# Supplementary material for: TMED3 promotes the progression and development of lung squamous cell carcinoma by regulating EZR
Source: Cell Death Dis. 2021 Aug 24;12(9):804. doi: 10.1038/s41419-021-04086-9 (PMC8385054; doi:10.1038/s41419-021-04086-9)
Supplement: Supplementary file 1 — Supplementary tables [file 41419_2021_4086_MOESM1_ESM.docx]

Table S1. The target sequences and shRNA sequences

| Gene | No. | Target sequence (5'-3') | shRNA sequences (5'-3') |
| --- | --- | --- | --- |
| TMED3 | Pbr10331-a | CTCTCACAAGACCGTCTACTT | AAGTAGACGGTCTTGTGAGAG |
| TMED3 | Pbr10331-b | CTCTCACAAGACCGTCTACTT | AAGTAGACGGTCTTGTGAGAG |
| EZR | Pbr12106-a | GGCCAAAGAAGCCCAGGATGA | TCATCCTGGGCTTCTTTGGCC |
| EZR | Pbr12106-b | GGCCAAAGAAGCCCAGGATGA | TCATCCTGGGCTTCTTTGGCC |
| EZR | Pb12107-a | GAGAGAAACCGTGGAGAGAGA | TCTCTCTCCACGGTTTCTCTC |
| EZR | Pb12107-b | GAGAGAAACCGTGGAGAGAGA | TCTCTCTCCACGGTTTCTCTC |
| EZR | Pbr12108-a | CGTGTACGAGCCGGTGAGCTA | TAGCTCACCGGCTCGTACACG |
| EZR | Pbr12108-b | CGTGTACGAGCCGGTGAGCTA | TAGCTCACCGGCTCGTACACG |
|  |  |  |  |

Table S2. Antibodies used in western blotting and IHC

| Antibody Name | Band Size (KDa) | Dilution | Antibody Source | Company | Catalog No. |
| --- | --- | --- | --- | --- | --- |
| TMED3 | 25 | 1:2000 | Rabbit | abcam | ab223175 |
| EZR | 69 | 1:2000 | Rabbit | abcam | ab40839 |
| Akt | 60 | 1:1000 | Rabbit | CST | 4685 |
| p-Akt | 60 | 1:1000 | Rabbit | Bioss | BS-5193R |
| Cyclin D1 | 36 | 1:2000 | Rabbit | CST | 2978 |
| CDK6 | 37 | 1:1000 | Rabbit | abcam | ab151247 |
| PIK3CA | 110 | 1:1000 | Rabbit | abcam | ab40776 |
| GAPDH | 37 | 1:3000 | Rabbit | Bioworld | AP0063 |

| Primary antibodies | Dilution in IHC | Source species | Company | Catalog No. |
| --- | --- | --- | --- | --- |
| Ki67 | 1:400 | Rabbit | abcam | ab6721 |
| TMED3 | 1:100 | Rabbit | abcam | ab223175 |
| EZR | 1:100 | Rabbit | abcam | ab179445 |
| Secondary antibody | Dilution |  | Company | Catalog No. |
| HRP Goat Anti-Rabbit IgG (WB) | 1:3000 |  | Beyotime | A0208 |
| HRP Goat Anti-Rabbit IgG (IHC) | 1:200 |  | Abcam | Ab111909 |

Table S3. Primers used in qPCR

| Primer Name | Upstream Primer  Sequence (5’-3’) | Downstream Primer  Sequence (5’-3’) |
| --- | --- | --- |
| TMED3 | GGCGTGAAGTTCTCCCTGGATT | GCTGTCGTACTGCTTCTTCGTTTC |
| EZR | AAGCTGGATAAGAAGGTGTCTGC | TGGGTGATGTCCTGGATGAG |
| GAPDH | TGACTTCAACAGCGACACCCA | CACCCTGTTGCTGTAGCCAAA |
| RPL31 | GCTCAACAAAGCTGTCTGGG | TTATGGGCTCTTGGCGACT |
| MAPK3 | ATTGTGCAGGACCTGATGGA | ACGTTGGCGGAGTGGATGTA |
| RPL37 | TCATCGTTTGGAAAGCGTCG | AGCCACATTTGCCACAGGTC |
| MAT2A | AGTTGTGCCTGCGAAATACC | TTGCGTCCAGTCAAACCAG |
| CCND2 | CATTTACACCGACAACTCCATC | GCTGCCAGGTTCCACTTCAA |
| EIF4B | CTTTTGGCCGTGATAGAAATCG | CATCAAAGCTGTCTGTAGCAGGA |
| EIF3L | GCGGGAGAAATATGGGGACA | TACAGGCGACAGGAACTTGG |
| EGFR | AGGCACGAGTAACAAGCTCAC | ATGAGGACATAACCAGCCACC |
| EIF5 | CGTATCCAGCAGTGAGACACCA | TCCATTCGACGCCTTTGAG |
| FOXO1 | ACCCAGCCCAAACTACCAA | ACTGACTCATACCTCCATAACTCG |
| SMAD3 | CACGCAGAACGTGAACACC | GGCAGTAGATAACGTGAGGGA |
| PPP2R1A | GAAAAGTTTGGGAAGGAGTGG | AGTCATGCGGTGCAGGTAGT |
| RPL27A | GCTTCTGCCCAACTGTCAAC | GCAGCCCCAGTCTTGTTTT |
| MRPS27 | GACAACCAGGGGTCAGAAAA | CGTTCCAGGTATTGAGGAAGC |
| FOS | CAGACTACGAGGCGTCATCC | TCTGCGGGTGAGTGGTAGTA |
| GJB4 | CAACGTCTGCTATGACGAGTTCT | CCGTGTTTCAGGTGGTGCTT |
| PNO1 | GGAACATTTGGGACTTCAGATACG | CCACCTGAAAGCCGAGAATAAA |
