# Supplementary material for: TMED3 promotes the progression and development of lung squamous cell carcinoma by regulating EZR
Source: Cell Death Dis. 2021 Aug 24;12(9):804. doi: 10.1038/s41419-021-04086-9 (PMC8385054; doi:10.1038/s41419-021-04086-9)
Supplement: Supplementary file 2 — Supplementary figure legends [file 41419_2021_4086_MOESM2_ESM.docx]

**Figure S1.** (A) Correlation analysis of TMED3 expression level and survival time of patients with LUSC. (B, C) The specificity and validity of the lentivirus-mediated shRNA knockdown of TMED3 expression in EBC-1, NCI-H520 and SK-MES-1 cells was verified by qPCR (B) and western blot (C).

**Figure S2.** (A) The effect of TMED3 downregulation on the expression of apoptosis-related proteins in NCI-H520 was studied using human apoptotic antibody array. (B) Effect of TMED3 downregulation on protein expression of EMT marker in NCI-H520 and SK-MES-1 cells.

**Figure S3.** (A) The enrichment of the DEGs in IPA disease and function was analyzed by IPA. (B) The enrichment of the DEGs in canonical signaling pathways was analyzed by IPA. (C) Interaction network diagram among DEGs was analyzed by IPA. (D) The significantly downregulated DEGs in the shTMED3 group for qPCR verification. (E) The most significantly downregulated candidate genes, including EGFR, EZR, GJB4 were selected for western blot analysis in NCI-H520 cells. (F) The protein levels of TMED3 and EZR of NCI-H520 cells in NC(OE+KD), TMED3+NC(KD), shEZR+NC(OE) and shEZR+TMED3 groups were detected by western blot.
