# Supplementary figures and images for: TMED3 promotes the progression and development of lung squamous cell carcinoma by regulating EZR
Source: Cell Death Dis. 2021 Aug 24;12(9):804. doi: 10.1038/s41419-021-04086-9 (PMC8385054; doi:10.1038/s41419-021-04086-9)

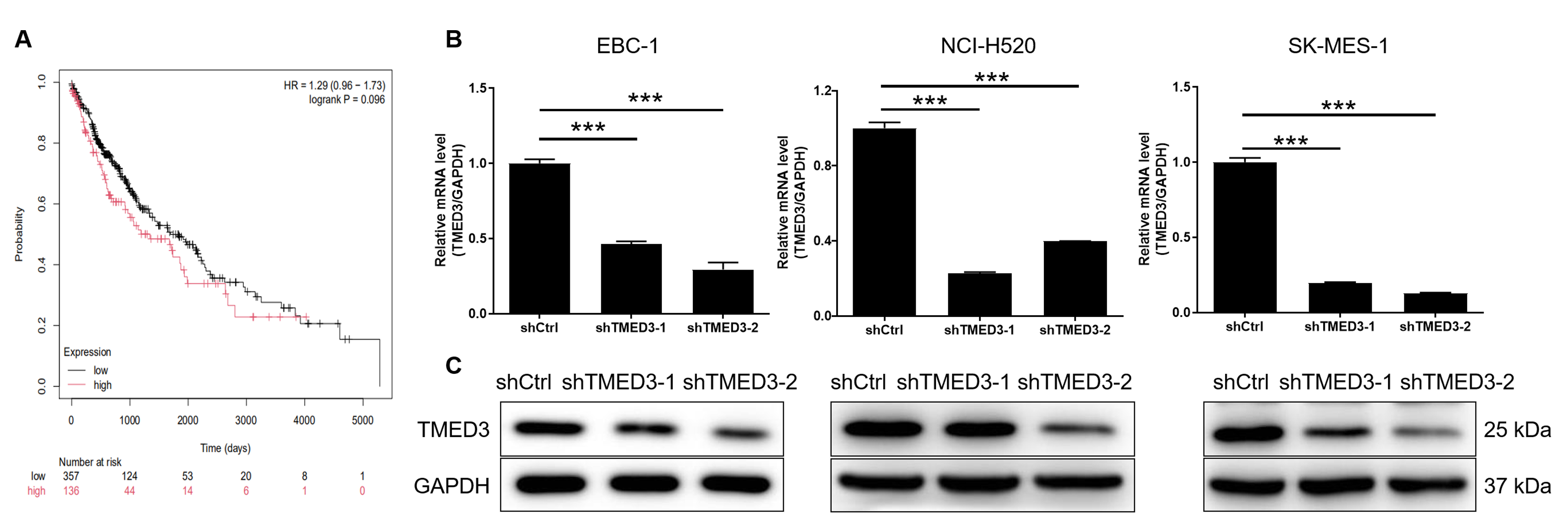

Supplement: Supplementary file 3 — Figure S1 [file 41419_2021_4086_MOESM3_ESM.tif]

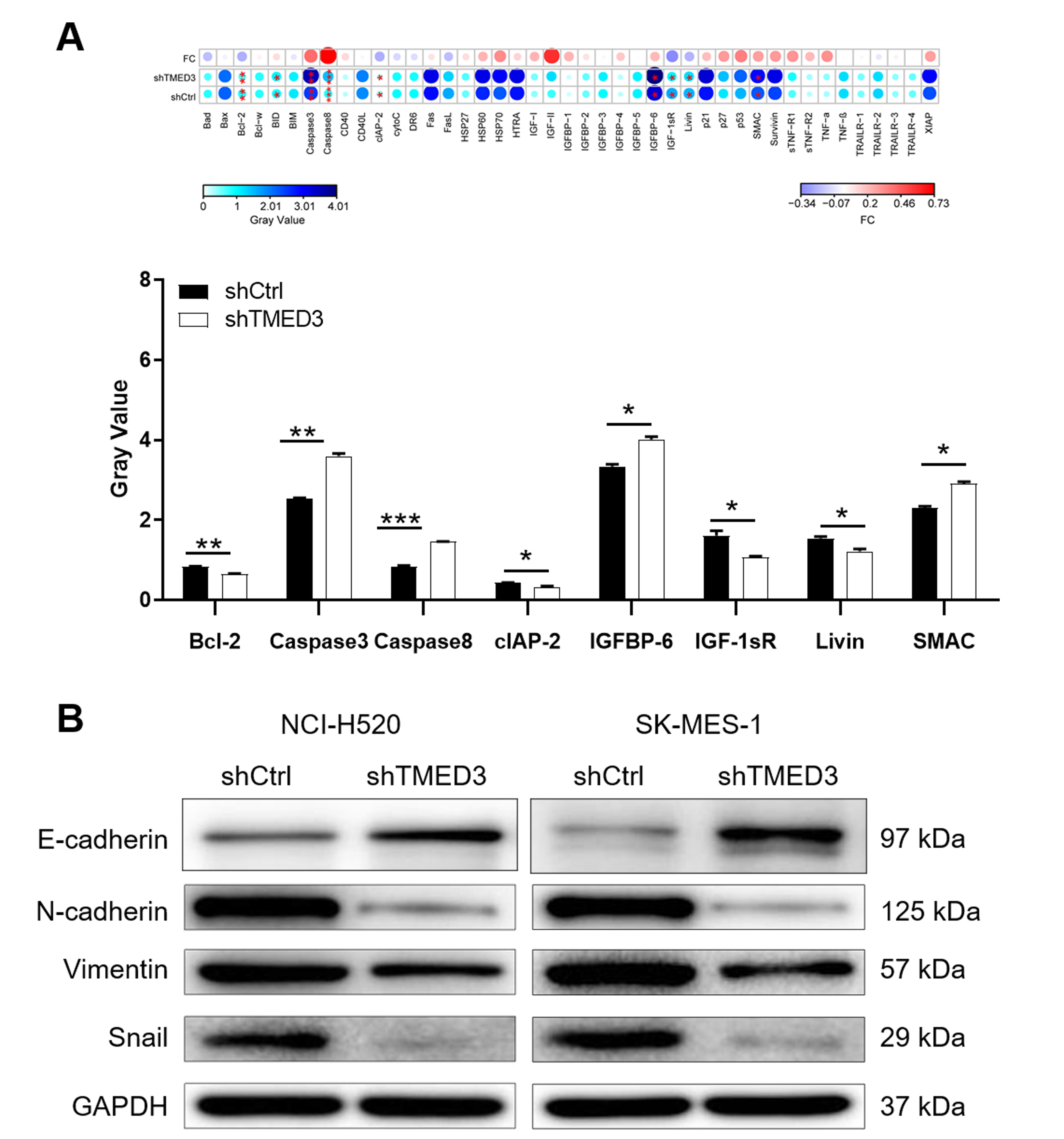

Supplement: Supplementary file 4 — Figure S2 [file 41419_2021_4086_MOESM4_ESM.tif]

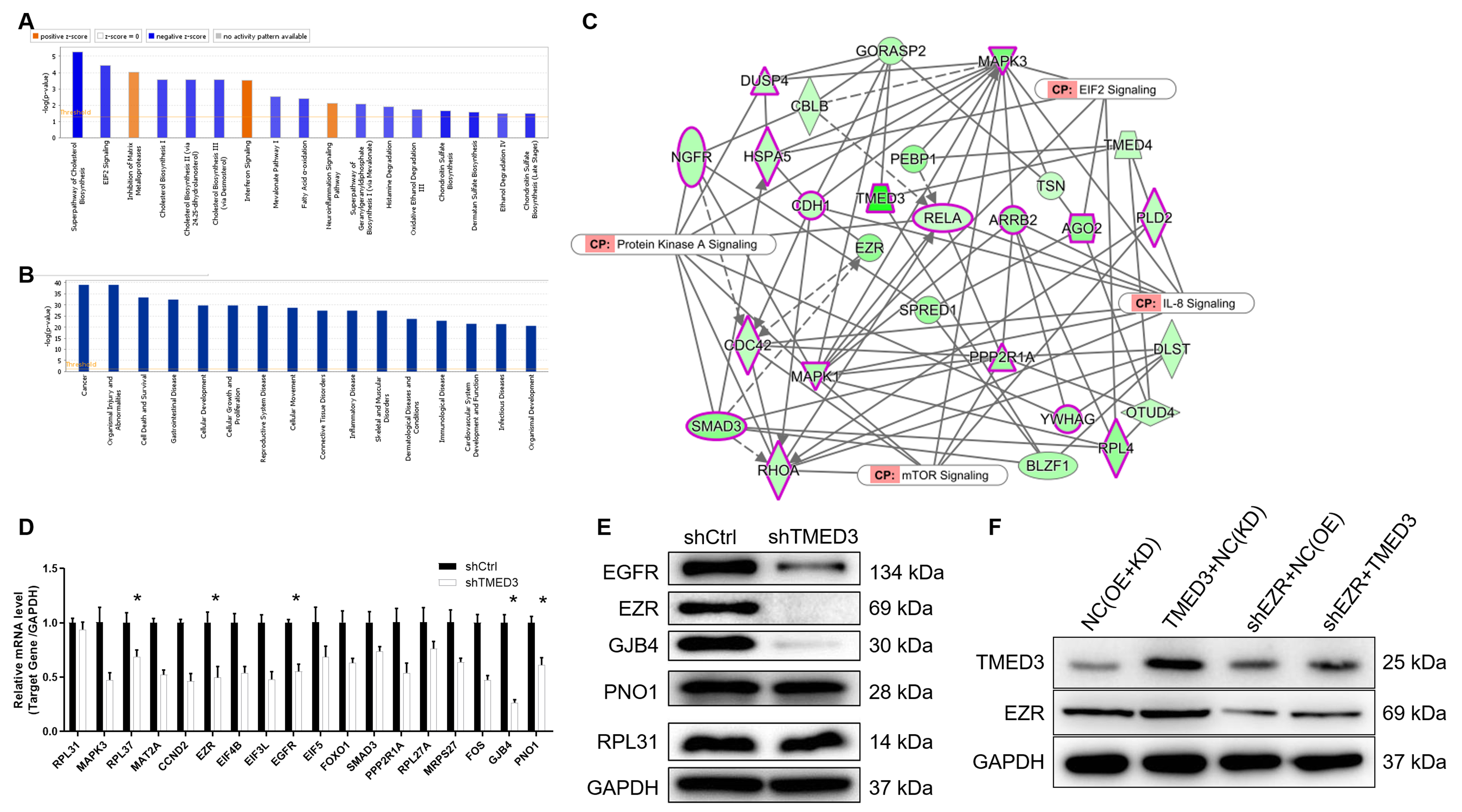

Supplement: Supplementary file 5 — Figure S3 [file 41419_2021_4086_MOESM5_ESM.tif]
