# Supplementary material for: TMED3 promotes the progression and development of lung squamous cell carcinoma by regulating EZR
Source: Cell Death Dis. 2021 Aug 24;12(9):804. doi: 10.1038/s41419-021-04086-9 (PMC8385054; doi:10.1038/s41419-021-04086-9)
Supplement: Supplementary file 7 — cddis-author-contribution-form [file 41419_2021_4086_MOESM7_ESM.pdf]

DECLARATION OF CONTRIBUTIONS TO ARTICLE

ADMC

Manuscript Number: CDDIS-21-0281

Journal Name: *Cell Death & Disease* (the 'Journal')

Proposed Title of the Contribution:

TMED3 promotes the progression and development of lung squamous cell carcinoma by regulating EZR (the 'Contribution)

Author(s):

An Xie, Xinping Xu, Peng Kuang, Ling Zhang, Feng Yu (the 'Authors')

For all *CDDis* articles, each person named as an author in the published version must be able to show he or she has contributed substantially to the article.

Authorship credit should be based on 1) substantial contributions to conception and design, acquisition of data, or analysis and interpretation of data; 2) drafting the article or revising it critically for important intellectual content; and 3) final approval of the version to be published. Authors should meet conditions 1, 2 and 3.

Any person who cannot be shown to have made a substantial contribution to the article cannot be listed as an author in the final version. The name of any person who is deemed to have made a minor contribution can, however, appear in the Acknowledgments section of the article.

Please complete the table below to indicate the contributions of all named authors to the manuscript.

Author Full Name:	Specification of Contribution to the Manuscript:
An Xie	operated the cell experiments and produced the manuscript
Xinping Xu	operated the cell experiments
Peng Kuang	performed animal experiments
Ling Zhang	conducted the data collection and analysis
Feng Yu	designed this program and revised the manuscript

Please complete the table below to indicate the contributions of all named authors to the figures.

Figure 1:
Ling Zhang, Feng Yu

Figure 2:
An Xie, Feng Yu

Figure 3:
Peng Kuang, Feng Yu

Figure 4:
Xinping Xu, Feng Yu

Figure 5:
An Xie，Xinping Xu, Feng Yu

Figure 6:

Signed for and on behalf of the Author(s):	Print Name:	Date:
Feng Yu	Feng Yu	2021.6.24
